# Oxygen Plasma Treated-Electrospun Polyhydroxyalkanoate Scaffolds for Hydrophilicity Improvement and Cell Adhesion

**DOI:** 10.3390/polym13071056

**Published:** 2021-03-27

**Authors:** Asiyah Esmail, João R. Pereira, Patrícia Zoio, Sara Silvestre, Ugur Deneb Menda, Chantal Sevrin, Christian Grandfils, Elvira Fortunato, Maria A. M. Reis, Célia Henriques, Abel Oliva, Filomena Freitas

**Affiliations:** 1 UCIBIO-REQUIMTE, Chemistry Department, Nova School of Sciences and Technology, 2829-516 Caparica, Portugal; a.esmail@campus.fct.unl.pt (A.E.); jra.pereira@campus.fct.unl.pt (J.R.P.); amr@fct.unl.pt (M.A.M.R.); 2 ITQB NOVA-Instituto de Tecnologia Química e Biológica António Xavier, Nova University Lisbon, 2780-157 Oeiras, Portugal; patricia.zoio@itqb.unl.pt (P.Z.); oliva@itqb.unl.pt (A.O.); 3 iBET, Instituto de Biologia Experimental e Tecnológica, 2780-157 Oeiras, Portugal; 4 CENIMAT/i3N, Materials Science Department, Nova School of Science and Technology, 2829-516 Caparica, Portugal; si.silvestre@campus.fct.unl.pt (S.S.); u.menda@fct.unl.pt (U.D.M.); emf@fct.unl.pt (E.F.); 5 CEIB-Interfaculty Research Centre of Biomaterials, University of Liège, B-4000 Liège, Belgium; csevrin@uliege.be (C.S.); c.grandfils@uliege.be (C.G.); 6 CENIMAT/i3N, Physics Department, Nova School of Sciences and Technology, 2829-516 Caparica, Portugal; crh@fct.unl.pt

**Keywords:** poly(hydroxyalkanoates), electrospinning, oxygen plasma, biocompatibility

## Abstract

Poly(hydroxyalkanoates) (PHAs) with differing material properties, namely, the homopolymer poly(3-hydroxybutyrate), P(3HB), the copolymer poly(3-hydroxybutyrate-co-3-hydroxyvalerate), P(3HB-co-3HV), with a 3HV content of 25 wt.% and a medium chain length PHA, and mcl-PHA, mainly composed of 3-hydroxydecanoate, were studied as scaffolding material for cell culture. P(3HB) and P(3HB-co-3HV) were individually spun into fibers, as well as blends of the mcl-PHA with each of the scl-PHAs. An overall biopolymer concentration of 4 wt.% was used to prepare the electrospinning solutions, using chloroform as the solvent. A stable electrospinning process and good quality fibers were obtained for a solution flow rate of 0.5 mL h^−1^, a needle tip collector distance of 20 cm and a voltage of 12 kV for P(3HB) and P(3HB-co-3HV) solutions, while for the mcl-PHA the distance was increased to 25 cm and the voltage to 15 kV. The scaffolds’ hydrophilicity was significantly increased under exposure to oxygen plasma as a surface treatment. Complete wetting was obtained for the oxygen plasma treated scaffolds and the water uptake degree increased in all treated scaffolds. The biopolymers crystallinity was not affected by the electrospinning process, while their treatment with oxygen plasma decreased their crystalline fraction. Human dermal fibroblasts were able to adhere and proliferate within the electrospun PHA-based scaffolds. The P(3HB-co-3HV): mcl-PHA oxygen plasma treated scaffold highlighted the most promising results with a cell adhesion rate of 40 ± 8%, compared to 14 ± 4% for the commercial oxygen plasma treated polystyrene scaffold Alvetex^TM^. Scaffolds based on P(3HB-co-3HV): mcl-PHA blends produced by electrospinning and submitted to oxygen plasma exposure are therefore promising biomaterials for the development of scaffolds for tissue engineering.

## 1. Introduction

A scaffold is a 3D structure made of synthetic, natural or mixed components that serves as support for cellular proliferation and differentiation, in view to mimic the microstructure, mechanical properties and biochemical functionality of living tissues [1,2,3]. This material should fulfil to several specifications before considering its medical application. Amongst other, it should be a biocompatible and non-immunogenic and should present an interconnected open-pore geometry with pore size allowing cell colonization, adhesion, growth and reorganization [1,4]. Moreover, mechanical properties should sustain the structure required for cell ingrowth and matrix formation [1,5]. Scaffolds made of a wide variety of materials, including natural and synthetic polymers, recombinant proteins, ceramics and metal composites. Some of them have been able to generate in vitro different biological tissue-like structures (blood vessels [6], skin [7], bone [8] and gut [9]) with potential applications in translational research, drug discovery and clinical transplantation or [3,10].

The selection of a scaffold architecture and material depends on the specific tissue engineering application [11]. Although synthetic materials, like synthetic polymers and bioactive glasses, are advantageous for their defined composition, the possibility they offer to tailor the scaffold mechanical properties and degradability, they may lack sites for cellular adhesion [12,13]. Natural materials, such as poly(hydroxyalkanoates) (PHAs), gelatin and chitosan, offer biocompatibility and cellular interaction, besides being biodegradable into non-toxic by-products [14,15,16,17]. However, being from natural origin, variability from batch to batch has been reported [3,11,18]. Fibrous scaffolds have attracted considerable attention because their fibers form a network that mimics the extracellular matrix (ECM) which is structurally convenient to promote cell adhesion, proliferation, differentiation and matrix deposition. Despite the large volume scaffolds being difficult to make, which limits this technique scale-up, electrospinning is a versatile technology giving rise to sub-micrometric and randomly oriented fiber networks with high porosity and interconnected pores, i.e., similar to the extracellular matrix (ECM) [19,20].

PHAs are biopolyesters synthesized as intracellular carbon and energy reserves by many bacteria and Archeae [21]. Mechanical properties of PHAs can range from stiff and brittle polymers, such as poly(3-hydroxybutyrate) P(3HB), to elastomers such as the medium-chain length PHAs (mcl-PHAs) [13,21,22]. P(3HB) is a highly crystalline (±70%) homopolymer with a melting temperature of 173–180 °C [23]. Poly(3-hydroxybutyrate-co-3-hydroxyvalerate), P(3HB-co-3HV), a copolymer of 3HB and 3HV monomers, has lower crystallinity (52–73%) and melting point (137–170 °C), conferred by the presence of 3HV monomers [23,24,25]. Mcl-PHAs are composed of 6–14 carbon atoms monomers (e.g., 3-hydroxyhexanoate (3HHx), 3-hydroxyoctanoate (3HO), 3-hydroxydecanoate (3HD)) and are mostly amorphous, with crystallinity degrees below 40% and have low melting temperatures (40–60 °C) [21,22].

PHAs characteristics, such as biocompatibility, biodegradability and customizability, make them suitable candidates for the fabrication of scaffolds intended for cell culture [22,23,24,25,26]. The fabrication of biocompatible PHA-based electrospun scaffolds has been reported for several PHAs, namely, P(3HB) [27,28], P(3HB-co-3HHx) [28], P(3HB-4HB-3HV) [29] and P(3HB-co-3HV) [30,31]. However, electrospun fibers of mcl-PHAs are tacky and sticky at room temperature, which leads to loss of shape on collection and makes the production of fibrous scaffolds unfeasible. Blending a mcl-PHA with a scl-PHA allowed to overcome this difficulty, resulting in electrospun fibers with improved mechanical properties and degradation rate when compared to those made of the scl-PHA alone [32]. A melt processing technique was employed for the preparation of a bionanocomposite of a scl-PHA with bacterial cellulose nanofibers, resulting in scaffolds with suitable physical and biological properties [33]. PHA-based scaffolds still suffer from a few limitations, including their intrinsic hydrophobicity that hinders their use for animal cell culture. Surface treatments (e.g., oxygen or CO_2_ plasma) are typically adopted to modify the surface properties of PHA-based materials [34,35,36,37,38,39], such as roughness, wettability and biocompatibility. These surface treatments are indeed introducing functional groups with oxygen atoms (e.g., grafted ester, carboxyl or carbonyl groups) [38], with significant improvement in terms of bioactivity [40].

In this work, PHA-based fibrous scaffolds were developed via electrospinning and tested as a platform for in vitro cell-culture. Different types of PHAs were tested, including P(3HB), P(3HB-co-3HV) and mcl-PHA and their corresponding blends. Furthermore, the electrospun scaffolds were exposed to oxygen plasma to render them higher hydrophilic character. The novel structures where then characterized for their physical, chemical and biological properties.

## 2. Materials and Methods

### 2.1. Biopolymers

The homopolymer P(3HB) and the copolymer P(3HB-co-3HV), with a 3HV content of 25 wt.%, were obtained by cultivation of *Cupriavidus necator* DSM 428 (purchased from DSMZ, the German Collection of Microorganisms and Cell Cultures, Braunschweig, Germany) with used cooking oil as carbon source, as described by Cruz et al. [41]. For the production of the copolymer, levulinic acid was used as co-substrate in a fed-batch mode, as described by Wang et al. [42]. An mcl-PHA composed of 64 wt.% 3HD, 16 wt.% 3HO, 12 wt.% 3HDd and 7 wt.% 3HTd was obtained by cultivation of *Pseudomonas chlororaphis* DSM 19603 (purchased from DSMZ, Braunschweig, Germany), using glycerol as carbon source, as described by Meneses et al. [43]. The biopolymers were extracted from the lyophilized biomass by using a Soxhlet extraction with chloroform (Sigma-Aldrich, ≥99.8%, Darmstadt, Germany) and purified by precipitation in ice-cold ethanol (Carlo Erba Reagents, Cornaredo, Italy), as described by Pereira et al. [44].

### 2.2. Preparation of PHA-Based Scaffolds

#### 2.2.1. Electrospinning

The biopolymers were dissolved in chloroform (Sigma-Aldrich, ≥99.8%, Darmstadt, Germany) at concentrations of 4 wt.%, for P(3HB) and P(3HB-co-3HV), or 12 and 25 wt.%, for the mcl-PHA. Blends of mcl-PHA with either P(3HB) or P(3HB-co-3HV) were prepared at an overall biopolymer concentration of 4 wt.%, with scl-/mcl-PHA adopting weight compositional ratios of 50:50, 60:40 and 70:30. The electrospinning apparatus consisted of a syringe pump (NE-1000 Programmable Single Syringe Pump, New Era PumpSystemsInc, Farmingdale, NY, USA), a high-voltage power supply (T1CP300304p, ISEG, Radeberg, Germany) and a homemade grounded slowly rotating collector. A 5 mL syringe containing the polymer solution was loaded into the syringe pump and a metallic blunt tip needle with an inner diameter of 0.508 mm was attached to the syringe. A voltage was applied to the needle by means of the high-voltage supply. The electrospun mats were collected on a grounded planar plate covered by an aluminum foil. Different electrospinning parameters were studied, namely, polymer concentration, voltage (8, 10, 12 and 15 kV), solution feeding rate (0.5 and 1.0 mL h^−1^) and needle-collector distance (20 and 25 cm). To monitor the result of the process according to the studied parameters, the fibers were collected for a few seconds onto a glass slide in contact with the aluminum foil. The glass slide was then observed under an optical microscope (Visiscope TL524PI, VWR, Alfragide, Portugal) equipped with a camera (Visicam3.0, VWR, Alfragide, Portugal). The experiments were performed at room temperature.

#### 2.2.2. Oxygen Plasma Treatment

**Figure 1:**
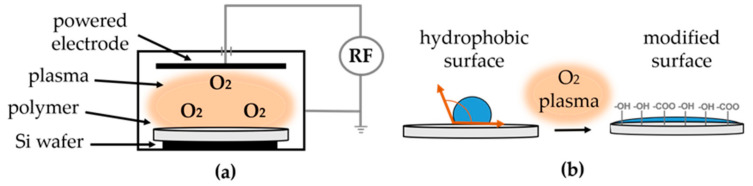
Schematic drawing of the oxygen plasma treatment (**a**) and the resulting modified surface (**b**).

The electrospun scaffolds were mounted on a silicon wafer with the aid of carbon tape. Samples were placed on a reactive ion etching (RIE) system (Trion Minilock Phantom III, Clearwater, FL, USA) and treated with oxygen plasma (Figure 1a). A plasma treatment of 12 min, at a pressure of 100 mTorr and O_2_ flow rate of 10 sccm was performed. A hydrophilic surface was obtained, as schematically shown in Figure 1b).

### 2.3. Characterization

#### 2.3.1. Molecular Mass Distribution of the Biopolymers

Number and weight average molecular weights, M_n_ and M_w_, respectively and the polydispersity index (PDI = M_n_/M_w_) of the samples were determined by a size exclusion chromatography (SEC) System (Waters Millenium, Milford, MA, USA), using monodisperse polystyrene standards (800–504,000 Da), as described by Pereira et al. [44].

#### 2.3.2. Thermal Analysis

Differential scanning calorimetry (DSC) analysis was carried out in a DSC25 Discovery Series (TA Instruments, New Castle, DE, USA) equipped with a cooling system, System 90 (TA Instruments, New Castle, DE, USA). The samples were placed in aluminum crucibles and analyzed in the temperature range between −90 ºC and 200 °C, at heating and cooling rates of 10 °C min^−1^ under N_2_ atmosphere. Three thermal cycles were performed. The melting temperature (*T_m_*, °C) was determined at the minimum of the endothermic peak. The crystallinity (*X_c_*, %) of the samples was estimated as the ratio between melting enthalpy (Δ*H_m_*, J g^−1^) of its melting peak and the melting enthalpy of a 100% crystalline P(3HB), earlier reported by Morais et al. [45] and equal to 146 J g^−1^. Thermogravimetric Analysis (TGA) was performed with a Labsys EVO (Setaram Instrumentation, France). Samples were placed in aluminium crucibles and analyzed in the temperature range between 25 and 500 °C, at 10 °C min^−1^.

#### 2.3.3. Scanning Electron Microscopy (SEM)

Samples of the electrospun scaffolds were mounted for SEM observation using double sided carbon tape and aluminum stubs and sputter coated with gold-palladium (60%/40%) alloy (Q150T ES, Quorum, Lewes, UK). The analysis was performed using a bench top scanning electron microscope (TM3030 Plus, Hitachi, Tokyo, Japan) using an acceleration voltage of 15 kV. The obtained SEM images were processed by ImageJ.

#### 2.3.4. Water Contact Angle and Water Uptake Degree

The water contact angle of the scaffolds was determined by the sessile drop method, as described by Rebocho et al. [46], using a CAM 200 goniometer (KSV Instruments Ltd., Espoo, Finland). For determination of the water uptake degree, scaffolds samples with a size of 1.0 × 1.0 cm^2^ were cut and weighted and their thickness was measured with a micrometer (Elcometer, Manchester, England). The samples were immersed in deionized water (15 mL), in closed vials that were kept at 30 °C, during 24 h. The water uptake degree of each sample was calculated with the following Equation:

(1) $$Water\;uptake\;degree\left(\%\right)=\frac{X2-X1}{X1}\times 100$$

where *X1* is the initial mass of the dry sample and *X2* its final mass after swelling. The samples thickness after immersion was also measured.

### 2.4. Biological Assays

#### 2.4.1. Cell Culture

Primary human dermal fibroblasts from neonatal foreskin (HDFn) (CellnTec, Bern, Switzerland) were maintained in Fibroblast Growth medium (FGM) composed of Iscove’s Modified Dulbecco’s Medium (IMDM) with Glutamax^TM^ (Thermo Fisher Scientific, Waltham, MA, USA) supplemented with 10% Fetal Bovine Serum (FBS, Thermo Fisher Scientific, Waltham, MA, USA) and 0.1% Penicillin-Streptomycin (Penstrep, Thermo Fisher Scientific, Waltham, MA, USA). HDFns were incubated at 37 °C, 5% CO_2_/95% humidified air in a cell culture incubator and subcultured at confluency of 90%, following supplier’s instructions. HDFns between passages 4 and 7 were used for all experiments for reproducibility purposes.

#### 2.4.2. Cell Seeding

The scaffolds were cut into circles of 1.5 cm in diameter to fit the wells of 24-well plates and sterilized under a 22-Watt UV lamp. Three biological replicates and three technical replicates for each biological replicate were performed. The assays were performed in triplicate. Inert polystyrene scaffolds (Alvetex^TM^, Reprocell Europe, Glasgow, UK) were pre-treated with 70% ethanol followed by two washes with PBS. HDFns (2 × 10^4^ cells) were seeded onto each scaffold and on empty wells (control) and maintained in FGM for 48 h at 37 °C in a 5% CO_2_/95% humidified incubator.

#### 2.4.3. Cell Viability (MTT Assay)

Cell viability was assayed using an MTT (thiazolyl blue tetrazolium bromide, Sigma-Aldrich) solution (5 mg mL^−1^ in PBS). The MTT solution (40 μL) was added to seeded scaffolds and the plates were incubated at 37 °C, for 4 h. After incubation, the MTT solution was removed and 200 μL of the extraction solution (89% isopropanol, 10% Triton-X, 1% HCl 0.37%) was added to the wells. The plates were agitated in an orbital shaker for 10 min (150 rpm) and incubated with the extraction solution for 2 h, at room temperature in the absence of light, to allow dissolution of formazan crystals. The content of each well was homogenized and 200 μm were transferred to a 96-well plate. The absorbance was measured at 570 nm. Triplicates of each type of scaffold were used in every assay. MTT assays were performed three times, in the same conditions, for statistical relevance. The results were submitted to statistical analysis with ordinary one-way ANOVA (GraphPad Prism 8.2.0, San Diego, CA, USA).

#### 2.4.4. Cell Morphology

To assess cell morphology, the cells were fixed with 2.5% glutaraldehyde (Carl Roth) for one hour, at room temperature. The scaffolds were subsequently washed with PBS (1×) and distilled water (2×), for 2 min and dehydrated with a graded ethanol in deionized water series (25%, 50%, 75%, 95% and 100%). Each exchange took 5 min, except the final one that was repeated twice, with a duration of 10 min. After drying in a fume hood, the scaffolds were stored in a desiccator until observation by SEM as described above.

## 3. Results and Discussion

### 3.1. Biopolymers Characterization

**Figure 2:**
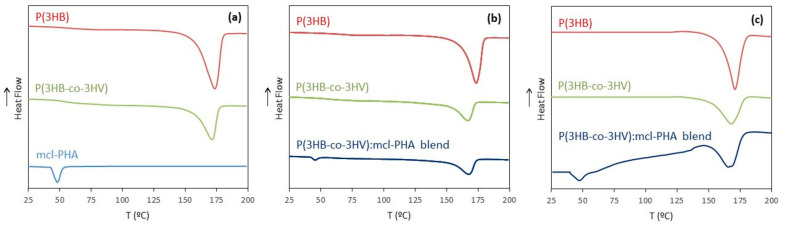
Differential scanning calorimetry (DSC) thermograms for (**a**) the untreated biopolymers, (**b**) the electrospun scaffolds and (**c**) the oxygen plasma treated electrospun scaffolds (red: P(3HB), green: P(3HB-co-3HV), light blue: mcl-PHA in (**a**), dark blue: P(3HB-co-3HV): mcl-PHA blend in (**b**,**c**)).

The homopolymer P(3HB) and the copolymer P(3HB-co-3HV) had similar M_w_ values (5.2 × 10^5^ and 5.6 × 10^5^ Da, respectively) (Table 1). P(3HB) had a polydispersity index (PDI) of 1.80, which is only slightly higher than that of P(3HB-co-3HV) (1.60). P(3HB) had a melting temperature (*T_m_*) of 173.5 °C (Figure 2a) and its thermal degradation (*T_deg_*) occurs at 293 °C (Table 1). The copolymer’s *T_m_* and *T_deg_* values were similar (171 and 292 °C, respectively) to those found for the homopolymer. P(3HB) melting enthalpy and crystallinity (76.6 J g^−1^ and 52.4%, respectively) were lower than those of P(3HB-co-3HV) (34.5 J g^−1^ and 23.6 %, respectively), which could be assigned to its 3HV content (25 wt.%).

The mcl-PHA, which was mainly composed of 3HD (64 wt%), with lower contents of 3HO (16 wt.%), 3HDd (12 wt.%) and 3HTd (7 wt.%), had a lower M_w_ (0.69 × 10^5^ Da) and lower PDI (1.50) than the scl-PHAs (Table 1). When compared to the scl-PHAs values (Table 1), the mcl-PHA’s Tm was considerably lower (48 °C), while the *T_deg_* was identical (292 °C). The biopolymer was also less crystalline than the scl-PHAs, with a melting enthalpy of 8.2 J g^−1^ and a crystallinity degree of 5.6%, all features readily explained by the presence of monomers with a high number of carbon atoms [47].

### 3.2. Scaffolds Preparation

#### 3.2.1. Electrospun Scaffolds Based on P(3HB), P(3HB-co-3HV) and mcl-PHA

Each biopolymer was dissolved in chloroform, which is one of the best solvents for PHAs [48]. Moreover, chloroform’s volatility allows for the complete solvent evaporation during the flight of the polymeric jet towards the collector, preventing the collection of a network of ribbon-shaped and fused fibers [49,50]. In order to successfully electrospin a solution, viscosity should be adequate: High enough to prevent the jet breaking or the formation beaded fibers but not too high to allow jet stretching. For a given solvent-polymer system, solution viscosity increases with both the polymer molecular mass and concentration and so adequate polymer concentrations may be not within a narrow range of values. Chloroform has been used in several studies to prepare PHAs solutions for electrospinning, with concentrations around 2–17 wt.%, for scl-PHAs [26,27,29,32] and 2–50 wt.% for mcl-PHAs [27,28,32]. In this study, a polymer concentration of 4 wt.% was used for the scl-PHAs, while for the mcl-PHA, higher concentrations (12 and 25 wt.%) were tested.

For preparation of the scaffolds, electrospinning process parameters were tested, namely, the needle tip collector distance, *d*, the solution flow rate, *ϕ* and the voltage applied to the needle, *V*, aiming at defining a set of parameters leading to regular shaped fibres [51]. For both P(3HB) and P(3HB-co-3HV) solutions, the distance was fixed at 20 cm and flow rates of 0.5 and 1.0 mL h^−1^ were tested for a voltage of 10 kV. For *d* = 20 cm and *ϕ* = 0.5 mL h^−1^, voltages were varied between 8 and 15 kV. At 8 kV, some bead formation was observed for P(3HB), which could be related to insufficient electric repulsion inside the polymeric jet able to stretch it, counteracting the force due to surface tension. On the other hand, for P(3HB-co-3HV), although smoother fibers were obtained, some blockage of the metallic tip occurred due to an unbalance between the solution feed rate and of the ability of the electric field to move the charged solution away from the tip of the needle (extracting rate). The accumulation of solution at the needle tip hinders the uninterrupted fiber formation. As the voltage was increased to 10, 12 and 15 kV, smoother fibers were obtained for both polymer solutions. However, the deposition of the fibers onto the collector was more stable at 12 kV. Increasing the flow rate to 1.0 mL h^−1^, for a voltage of 10 kV, resulted in unstable electrospinning process, indicating that the feeding rate was higher than the electric field extraction rate. The parameters that lead to a stable process and good quality fibers were selected to electrospin both solutions (Table 2).

Contrary to P(3HB) and P(3HB-co-3HV), no fibers were obtained from the mcl-PHA solution, which could be related to the lower viscosity of the solutions. However, even at concentrations of 12 and 25 wt.% it was not possible to obtain fibers and only dots of polymer could be observed at the collector. This difficulty in obtaining electrospun mcl-PHA fibers has been reported by Li et al. [32] that suggested it was due to the lower molecular weight of mcl-PHA, as well as to the branching of the polymer, which can hinder chain entanglement. Blending mcl-PHA with other biocompatible polymers or scl-PHAs is the most common strategy to obtain fibers that incorporate mcl-PHA, while enabling fiber formation [27,28,32,52].

#### 3.2.2. Electrospun Scaffolds Based on scl-/mcl-PHA Blends

P(3HB): mcl-PHA and P(3HB-co-3HV): mcl-PHA solutions in chloroform at different weight blend ratios (50:50, 60:40 and 70:30), with an overall polymer concentration of 4 wt.%, were tested to produce fibers by electrospinning. Processing parameters were fixed at the following values: *d* = 25 cm, *V* = 15 kV and *ϕ* = 0.5 mL h^−1^.

**Figure 3:**
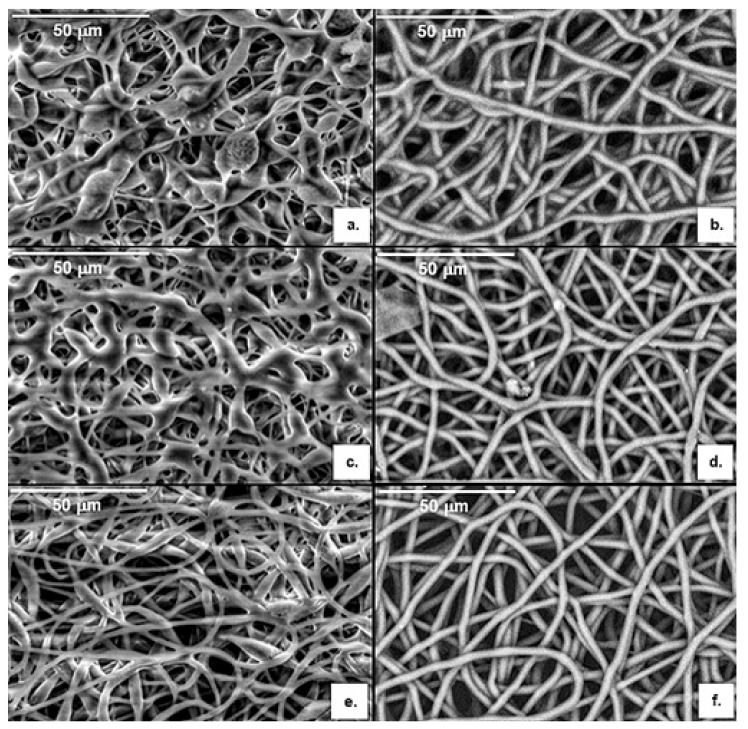
Images obtained by Scanning Electron Microscopy (SEM) analysis of the electrospun P(3HB): mcl-PHA fibers at blend ratios of 50:50 (**a**), 60:40 (**c**) and 70:30 (**e**) and the electrospun P(3HB-co-3HV): mcl-PHA fibers at blend ratios of 50:50 (**b**), 60:40 (**d**) and 70:30 (**f**).

For P(3HB): mcl-PHA blend ratios of 50:50 and 60:40, beaded-fibers were obtained (Figure 3a,c). This may be due to a relatively high surface tension and low viscosity of the spinning solutions. As the blend ratio increased to 70:30, fibers became much smoother (Figure 3e). Comparable results were obtained by Azari et al. [51], for blends of P(3HB) and a palm oil-based mcl-PHA, dissolved in a mixture of chloroform and dimethylformamide (DMF). However, bead-less fibers where only produced with a P(3HB): mcl-PHA ratio of 80:20, a lower mcl-PHA incorporation when compared to that in our study.

For the copolymer blends with the mcl-PHA, smooth bead-free microfibres were obtained for all tested ratios (Figure 3b,d,f). However, the electrospun mats had the greatest uniformity at a ratio of 70:30 (Figure 3f). Similar results were reported by Li et al. [32] that tested different blend solutions of the copolymer P(3HB-co-3HV) (25 mol% HV content) with the mcl-PHA P(HO-co-HHx) (92.6 mol% HO content) dissolved in a mixture of chloroform and DMF and obtained smooth, bead-free fibers from blend solutions at ratios above 65:35, with the greatest uniformity observed at a ratio of 75:25.

Together with P(3HB) and P(3HB-co-3HV), the P(3HB-co-3HV): mcl-PHA blend at a ratio of 70:30 was chosen for producing scaffolds, as indicated in Table 2, to be subject to the oxygen plasma treatment.

#### 3.2.3. Oxygen Plasma Treated Scaffolds

The electrospun scaffolds obtained from P(3HB), P(3HB-co-3HV) and P(3HB-co-3HV)/mcl-PHA blend solutions were exposed to oxygen plasma at a pressure of 100 mTorr and a flow rate of 10 sccm. This treatment was tested as a surface modification method aiming to increase the scaffolds’ hydrophilicity and, thus, facilitate cell adhesion. Plasma treatment with different fluids, including oxygen, nitrogen and carbon dioxide, were reported to render PHAs cast films more biocompatible by enhancing hydrophilicity and cell attachment [35,37,53,54].

### 3.3. Scaffolds Characterization

#### 3.3.1. Morphology

**Figure 4:**
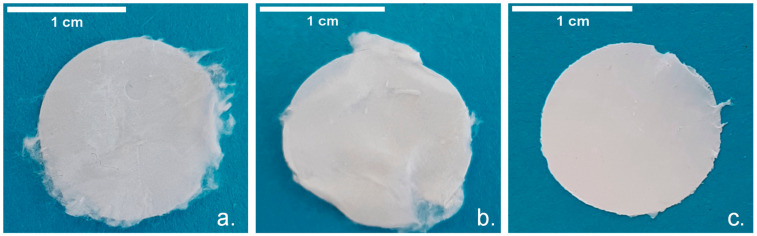
Electropsun (**a**) P(3HB), (**b**) P(3HB-co-3HV) and (**c**) P(3HB-co-3HV): mcl-PHA scaffolds.

**Figure 5:**
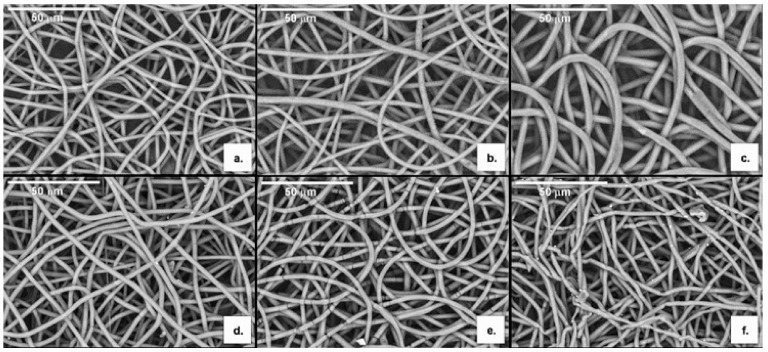
Images obtained by Scanning Electron Microscopy (SEM) analysis of the electrospun P(3HB), P(3HB-co-3HV) and P(3HB-co-3HV): mcl-PHA (70:30) blend scaffolds (**a**–**c**, respectively); SEM imaging of the electrospun fibers after 12 min of oxygen plasma exposure (**d**–**f**, respectively).

The electrospun P(3HB), P(3HB-co-3HV) and P(3HB-co-3HV): mcl-PHA scaffolds (Figure 4) were all similar in terms of texture, color and opacity, displaying a white and silky appearance and a thickness of 84 ± 21, 38 ± 10 and 39 ± 2 µm, respectively. Treatment with oxygen plasma had no visible impact on the macroscopic structures but some distinct features could be identified by SEM analysis (Figure 5).

The SEM images of the electrospun P(3HB), P(3HB-co-3HV) and P(3HB-co-3HV): mcl-PHA scaffolds show that all present smooth, bead-less branched and randomly organized fibers with high void space interconnectivity (Figure 5). The diameter of at least 30 fibers of each scaffold was measured with ImageJ. The P(3HB) scaffold (Figure 5a) presented fiber diameters of 1.7–3.4 µm and a mean diameter of 2.6 ± 0.4 µm, while the P(3HB-co-3HV) microfibers (Figure 5b) had slightly larger dimensions, with diameters ranging between 2.1 and 3.5 µm, with a mean diameter of 2.7 ± 0.4 µm. These values are comparable to that reported by Volova et al. [27] (2.5 µm) for a for a P(3HB-co-3HV) with 4.5–8% 3HV content. Sombatmankhong et al. [26] also reported similar values (2.3 ± 2.1 µm) for a P(3HB-co-3HV) (5% 3HV content) electrospun scaffold but higher values for P(3HB) electrospun fibers (3.7 ± 1.7 µm). On the other hand, Azari et al. [51] obtained P(3HB) fibers of smaller dimensions (1.3 ± 0.3 µm). Nevertheless, these reported values are merely indicative since fiber diameter is dependent on electrospinning parameters (including solution parameters and processing parameters). The diameter of the fibers of the P(3HB-co-3HV): mcl-PHA (70:30) blend scaffold (Figure 5c) is considerably higher than that in the scl-PHAs scaffolds, ranging between 3.6 and 4.6 µm, with a mean diameter of 4.0 ± 0.2 µm. These values are significantly higher than those of the scl-/mcl-PHA electrospun scaffolds reported for fibers of P(3HB-co-3HV) (25 mol% 3HV content) blended with PHOHHx (92.6 mol% 3HO) (0.48–0.76 µm range) [26] and for the blend of P(3HB) with mcl-PHA (0.81–1.31 µm) [52]. All the fibers obtained in the present study have a narrow size distribution, showing their size homogeneity.

After oxygen plasma exposure, alterations in the morphology of the fibers were noticed (Figure 5d–f). Regarding the P(3HB) and P(3HB-co-3HV) scaffolds, fractures along the fibers are visible (Figure 5d,e), especially in the copolymer scaffold, for which a higher number of said fractures is observed (Figure 5e). The fibers’ mean diameter was not affected in the P(3HB) scaffold, remaining the same after treatment (2.6 ± 0.5 µm), despite of a slight increase of their size distribution (1.4–3.6 µm). In contrast, a decrease in mean fiber diameter from 2.7 ± 0.4 to 2.2 ± 0.5 µm was observed for the P(3HB-co-3HV) scaffold and thinner fibers were also observed, with a wider fiber size distribution (1.1–3.5 µm). The fibers of the P(3HB-co-3HV): mcl-PHA scaffold, on the other hand, did not seem to suffer for much fracturing (Figure 3f), although a slight thinning was evidenced with a decrease in the fiber mean diameter from 4.0 ± 0.2 to 2.6 ± 0.2 µm. There was also a considerable broadening of the fibers size distribution (1.2–3.6 µm). These results show that subjecting the scaffolds to plasma treatment impacted the fibers homogeneity, which may be explained by the physical etching phenomena, where the ions in the plasma cause erosion of the material [36]. Nevertheless, the thinning of the fibers corresponded to an increase of the surface area, which might turn beneficial for promoting cell attachment [55,56].

Regarding the scaffolds’ porosity, no significant changes were noticed for the average pore size of the oxygen plasma treated scaffolds compared to the electropsun scaffolds (Table 3).

#### 3.3.2. Molecular Mass Distribution

A slight decrease of the biopolymers’ M_w_ upon exposure to the electrospinning conditions has been also observed (Table 1). The M_w_ of the P(3HB) electrospun fibers was 4.20 × 10^5^ Da, compared to 5.20 × 10^5^ Da of the raw biopolymer. Similarly, the M_w_ of the P(3HB-co-3HV) fibers showed a similar decrease from 5.60 × 10^5^ Da to 4.10 × 10^5^ Da. This drop in M_w_ of these aliphatic polyesters can be assigned to a partial hydrolysis of their ester bonds during the fabrication process and/or their storage. Interestingly, the biopolymer PDI was not significantly affected during electrospinning (Table 1). Exposure of the P(3HB) and P(3HB-co-3HV) scaffolds to oxygen plasma seems to provoke a further decrease in the biopolymers’ M_w_ but with an increase in PDI (Table 1). The M_w_ was reduced from 4.20 × 10^5^ and 4.10 × 10^5^ Da, respectively, to 3.50 × 10^5^ Da, while their PDI increased from 1.63–1.69 to 1.90–2.00. This increase in polydispersity can be explained by the surface character of plasma exposure. Indeed, polymer chains present at the surface of the fibers should be more prompted to hydrolysis than polymer chains embedded in their core.

An average M_w_ of 3.50 × 10^5^ Da was observed for the P(3HB-co-3HV) and mcl-PHA in the blend fibers (Table 1). This value is within the those obtained for the copolymer’s fibers (4.10 × 10^5^ Da) and those of the mcl-PHA (0.69 × 10^5^ Da). On the other hand, the PDI was higher than either biopolymer, 2.79 (Table 1), reflecting the higher size dispersity of the two biopolymer molecules present in the blend. Contrary to the scl-PHAs scaffolds, no reduction of the M_w_ was observed for the oxygen plasma treated P(3HB-co-3HV): mcl-PHA scaffolds and the PDI was only slightly increased from 2.8 to 2.9, suggesting that the PHA blend was more resistant to depolymerization by exposure to oxygen plasma than the single scl-PHA-based materials.

#### 3.3.3. Thermal Properties

The biopolymers’ thermal properties were not significantly changed after processing into microfibers nor more than by exposure to oxygen plasma (Table 1). For both P(3HB) and P(3HB-co-3HV) electrospun scaffolds, only a slight decrease of their *T_m_*, from 176 to 173 °C and from 173 to 167 °C, respectively, was noticed compared to the raw materials. Their subsequent oxygen plasma treatment did not cause any further decease (Table 1). This reduction in *T_m_* can be correlated to the M_w_ decline and differences in crystallinity observed after processing by electrospinning [20]. The lower *T_m_* of crystals made from lower-molecular-weight polymers can be explained by their higher content in chain ends [57]. Interestingly, the *T_deg_* was not altered either after electrospinning, either after oxygen plasma exposure, all samples displaying values within 288–293 °C (Table 1).

A slight increase of the Δ*H_m_* and the *X_c_* were noticed for the scl-PHAs scaffolds compared to the unprocessed biopolymers (Table 1). The Δ*H_m_* increased from 76.5 to 77.5 J g^−1^, for the electrospun P(3HB) scaffold and from 34.5 to 36.1 J g^−1^, for the electrospun P(3HB-co-3HV) scaffold. Concomitantly, the *X_c_* increased from 52.4% to 53.1% and from 23.6% to 24.7%, for the homopolymer and the copolymer, respectively. The rise in crystallinity can be attributed to the orientation of macromolecular chains in the longitudinal fiber direction during the electrospinning process, that may have promoted crystallization [58]. On the other hand, exposure to oxygen plasma had a much more relevant impact on the biopolymers, as shown by the considerably lower Δ*H_m_* and *X_c_* values (Table 1). These results demonstrate that the treatment led to more exposed amorphous phases in the biopolymers.

For the P(3HB-co-3HV): mcl-PHA blend, two melting temperatures (169 and 46 °C) were detected (Table 1), one corresponding to the *T_m_* of P(3HB-co-3HV) and the other to the *T_m_* of the mcl-PHA in the composite, demonstrating a successful blend development. The degradation temperature (290 °C) (Table 1) was close to the value for unprocessed mcl-PHA and P(3HB-co-3HV) (both 292 °C).

#### 3.3.4. Water Contact Angle and Water Uptake Degree

**Figure 6:**
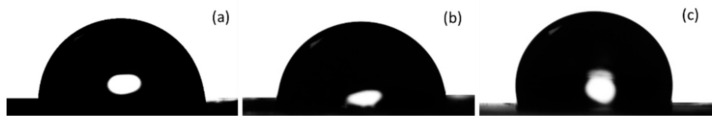
Images of the water droplets used for contact angle measurements of the electrospun scaffolds: (**a**) P(3HB), (**b**) P(3HB-co-3HV) and (**c**) P(3HB-co-3HV): mcl-PHA blend.

The P(3HB-co-3HV) and the scl-/mcl-PHA electrospun scaffolds presented water contact angles of 96.8 ± 1.3° and 113.5 ± 0.7°, respectively (Table 3), which demonstrates their hydrophobicity. On the other hand, the P(3HB) electrospun scaffold was hydrophilic, given its water contact angle (84.3 ± 1.8°) was below 90° [59]. The images of water droplets used for contact angle measurements are shown in Figure 6. Water contact angles in the range 115–126° have been reported for PHA-based electrospun scaffolds, including P(3HB), P(3HB-co-3HV) and their blends [26,60]. The lower values observed for the P(3HB) and P(3HB-co-3HV) scaffolds may be related to the higher surface roughness of the produced microfiber meshes that lead to a decrease of the water drop-material contact area, thus increasing the water contact angle of the materials [61].

After exposure to oxygen plasma, all three scaffolds experienced complete wetting (θ = 0°) (Table 3), thus showing that they were highly hydrophilic. It was not possible to collect images of water droplets because they were immediately absorbed by the scaffolds. The hydrophilic nature of the treated scaffolds was confirmed by measuring their water uptake degree (Table 3). Interestingly, the P(3HB) electrospun scaffold, which had no water uptake, displayed a water uptake degree of 294% after the oxygen plasma treatment. The water uptake degree of the P(3HB-co-3HV) scaffold also increased significantly upon exposure to oxygen plasma, from 77% to 205%. Although there was a change in mass due to water absorption, there was no changes in scaffold thickness or shape. Blending the copolymer with the mcl-PHA reduced the scaffolds ability to uptake water. Indeed, 0% water uptake degree was noticed on the P(3HB-co-3HV): mcl-PHA scaffold, compared to the P(3HB-co-3HV) scaffold (77%). This represents an increase of 17% after exposure to oxygen plasma, which was nevertheless still significantly lower than the value found for the treated copolymer scaffold (205%). Overall, the oxygen plasma treatment proved to not only enhance surface hydrophilicity and, therefore, facilitate also water diffusion, which is crucial for 3D-cell culture scaffolds.

#### 3.3.5. Fourier Transform Infrared Spectroscopy

**Figure 7:**
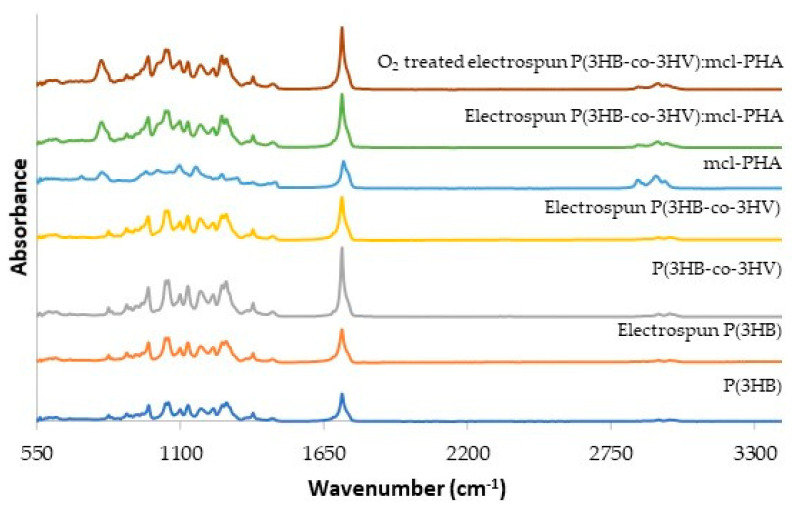
FTIR-ATR spectra of the biopolymers P(3HB), P(3HB-co-3HV) and the mcl-PHA, of the electrospun scaffolds made of P(3HB), P(3HB-co-3HV) and the P(3HB-co-3HV): mcl-PHA blend and of the oxygen plasma treated P(3HB-co-3HV): mcl-PHA blend scaffold.

The FT-IR spectra for the biopolymers and the corresponding electrospun scaffolds are shown in Figure 7. In all spectra, an intense peak can be observed at around 1720–1750 cm^−1^, corresponding to the stretching band of the ester carbonyl group [62]. The bands between around 960 and 1280 cm^−1^, which are related to the degree of crystallinity, are significantly more intense for P(3HB) and P(3HB-co-3HV)) than for the mcl-PHA, in accordance with the biopolymers crystallinity degree (52.4% and 23.6%, for the scl-PHAs, respectively and 5.6% for the mcl-PHA) (Table 1). On the other hand, the bands near 2961–2854 cm^–1^ that correspond to the asymmetric CH_2_ of the lateral monomeric chains (the peak near 2924 cm^–1^), the methylene C-H elongation vibration (the peak near 2900 cm^–1^) and the symmetrical methyl group (the peak near 2847 cm^–1^) [63], are stronger for the mcl-PHA and weaker for the scl-PHAs. A peak near 800 cm^–1^ is also noticed in the mcl-PHA spectrum but not on the scl-PHAs spectra (Figure 7). The FTIR spectrum for the P(3HB-co-3HV): mcl-PHA blend combine the characteristic peaks for the co-polymer and the mcl-PHA.

Processing the biopolymers into electrospun fibers and, afterwards, subjecting them to oxygen plasma treatment, had no significant impact on the spectra as all the characteristic peaks were retained, with no relevant change in intensity (Figure 7).

### 3.4. Biological Assays

#### 3.4.1. Cell Viability

In order to assess the scaffolds functionality and biocompatibility, human dermal fibroblasts (HDFn) colonization, adhesion and viability were studied using the MTT assay on the electrospun meshes made from P(3HB), P(3HB-co-3HV) and P(3HB-co-3HV): mcl-PHA blend. As positive control, a commercial polystyrene scaffold (Alvetex™) commonly used for 3D cell culture of mammalian cells [64,65,66], was used as a reference.

**Figure 8:**
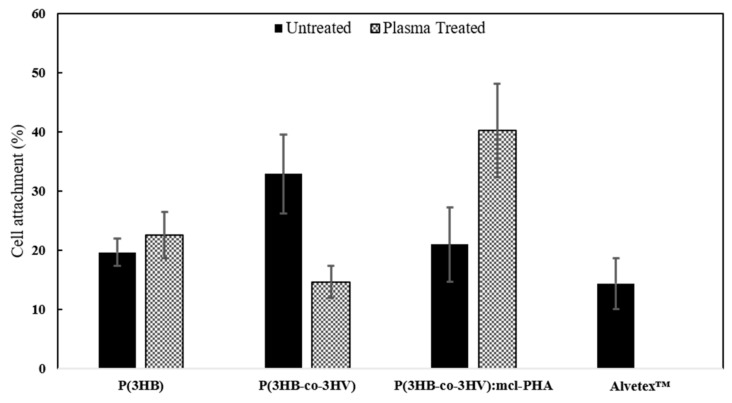
Comparison of the HDFns adhesion on different types of PHA based scaffolds with and without oxygen plasma treatment, measured by MTT assay. A commercial 3D mesh, Alvetex™, has been used as reference. The cell attachment values are the ratio between the absorbance of each sample and the attachment attained in polystyrene wells.

Three independent assays were carried out with HDFns of different passage for reproducibility purposes. Polystyrene wells were also used as additional positive control and referred as 100% attachment. A ratio between the absorbance of each scaffold and the control was calculated for every trial and then the average between all trials, as well as standard deviation was calculated (Figure 8). One-way ANOVA statistical method was utilized to compare the viability of HDFns cultured onto the different scaffolds and the results proved to be statistically significant (*p* < 0.05) with a *p*-value of 0.01.

Exception made of the electrospun P(3HB-co-3HV) plasma treated scaffold, which disclosed similar cell density (15 ± 3%) to Alvatex™ (14 ± 4%), all other polyester meshes (plasma treated and untreated) presented superior cell attachment values (Figure 4). The higher cell density values were noticed for the plasma treated P(3HB-co-3HV): mcl-PHA and the untreated P(3HB-co-3HV) scaffolds, whit improved cell attachment 2.8-fold and 2.3-fold. It is worth mentioning that the oxygen plasma treatment affected differently each scaffold. The homopolymer scaffold’s cell attachment only improved slightly upon the treatment, from 19.6 ± 2.3% to 22.5 ± 4.0%, while that of the P(3HB-co-3HV): mcl-PHA scaffold doubled its values (from 20.9 ± 6.3% to 40.2 ± 7.9%). For the P(3HB-co-3HV), on the other hand, a contrasting behavior was observed: the plasma treatment caused a reduction of cell attachment from 32.9 ± 6.6% to 14.6 ± 2.6%.

The lower values observed for the treated P(3HB-co-3HV) scaffold may be related to the damage noted in the fibers (revealed by SEM) after exposure to oxygen plasma (Figure 3e) that might have negatively impacted cell attachment, fiber heterogeneity has been reported to cause lower attachment of NIH 3T3 fibroblasts [55]. The P(3HB-co-3HV): mcl-PHA blend’s higher attachment after oxygen plasma treatment can be related to both increased surface hydrophilicity and thinning of the fibers. Thinner fibers have augmented surface area for adsorption of extracellular matrix proteins that interact with anchorage dependent mammalian cells and lead to improved attachment [67]. MG-63 cells and NIH 3T3 fibroblasts have shown superior attachment to smaller diameter fibers of biomaterials when compared to their larger diameter counterparts [56,68].

#### 3.4.2. Cell Morphology

**Figure 9:**
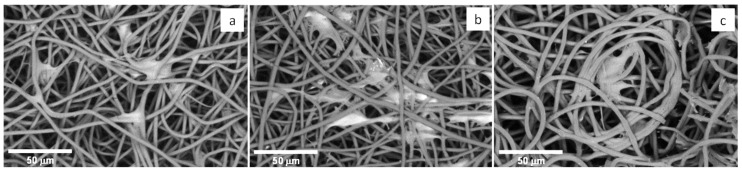
SEM images of HDFn attached to PHA-based electrospun scaffolds: (**a**) P(3HB), (**b**) P(3HB-co-3HV) and (**c**) P(3HB-co-3HV): mcl-PHA.

The cells’ morphology on the different scaffolds was observed under SEM. Noticeable cell attachment was observed for all fibrous scaffolds (Figure 9), proving them to be a favorable environment for cell adhesion and confirming MTT results. The fibroblasts present already an elongation of their shape in direction of the fiber axis stretching to adhesion points on adjacent fibers. This cell behavior is similar to other observations reported earlier involving fibroblast culture onto electrospun fibers, for example for mouse fibroblast cells (L929) cultured onto a P(3HB)/silk fibroin composite nanofiber mats [69].

## 4. Conclusions

This study demonstrated the suitability of the biopolyesters P(3HB), P(3HB-co-3HV) and an mcl-PHA for the preparation of electrospun scaffolds based with improved hydrophilicity and cell adhesion by exposure to oxygen plasma. The developed scaffolds possessed a porous structure, with interconnected pores and were hydrophilic. All scaffolds were biocompatible and provided an appropriate structure for cell adhesion. The most promising result was achieved for the oxygen plasma treated electropsun P(3HB-co-3HV): mcl-PHA blend scaffold, which displayed considerably higher cell attachment than the commercial synthetic material. Therefore, these original results encourage the future investigations on plasma treatment as a technique for developing hydrophilic electrospun scaffolds P(3HB-co-3HV): mcl-PHA scaffolds. Such biocompatible structures may find application in the development of 3D in vitro cell models, as well as for their consideration as degradable and biocompatible scaffold for tissue engineering applications.

## Figures and Tables

**Table 1:** Molecular mass distribution and thermal properties of the biopolymers P(3HB), P(3HB-co-3HV) and the mcl-PHA, as well as those of the scaffolds obtained by electrospinning and after oxygen plasma treatment (M_w_, molecular weight; PDI, polydispersity index; *T_m_*, melting temperature; *T_deg_*, degradation temperature; *X_c_*, crystallinity fraction; Δ*H_m_*, melting enthalpy).

Processing/Material	M_w_ (Da)	PDI	*T_m_* (°C)	*T_deg_* (°C)	∆*H_m_* (J g^−1^)	*X_c_* (%)
**None**						
P(3HB)	5.20 × 10^5^	1.80	176	293	76.5	52.4
P(3HB-*co*-3HV)	5.60 × 10^5^	1.60	171	292	34.5	23.6
mcl-PHA	0.69 × 10^5^	1.50	48	292	8.2	5.6
**Electrospinning**						
P(3HB) scaffold	4.20 × 10^5^	1.63	173	289	77.5	53.1
P(3HB-*co*-3HV) scaffold	4.10 × 10^5^	1.68	167	288	36.1	24.7
P(3HB-*co*-3HV): mcl-PHA blend scaffold	3.50×10^5^	2.79	168/46	290	30.5	20.9
**Electrospinning + O_2_ Plasma Treatment**						
P(3HB) scaffold	3.50 × 10^5^	1.90	171	291	56.6	38.8
P(3HB-*co*-3HV) scaffold	3.50 × 10^5^	2.00	168	293	26.3	18.0
P(3HB-*co*-3HV): mcl-PHA blend scaffold	3.50 × 10^5^	2.90	168/46	293	14.9	10.2

**Table 2:** Parameters selected to electrospin the biopolymers’ solutions.

Solution	*d* (cm)	*ϕ* (mL h^−1^)	*V* (kV)
P(3HB)	20	0.5	12
P(3HB-*co*-3HV)	20	0.5	12
P(3HB-*co*-3HV): mcl-PHA blend	25	0.5	15

**Table 3:** Water contact angles and water uptake degree of the scaffolds fabricated with P(3HB), P(3HB-co-3HV) and P(3HB-co-3HV): mcl-PHA blends.

Processing/Scaffold	Pore Size (µm)	Water Contact Angle (ϴ)	Water Uptake Degree (%)
**Electrospinning**			
P(3HB)	9.4 ± 3.5	84.3 ± 1.8	0
P(3HB-*co*-3HV)	10.2 ± 2.7	96.8 ± 1.3	77
P(3HB-*co*-3HV): mcl-PHA blend	10.2 ± 3.3	113.5 ± 0.7	0
**Electrospinning + O_2_ Plasma Treatment**			
P(3HB)	10.0 ± 2.9	0	294
P(3HB-*co*-3HV)	9.5 ± 2.5	0	205
P(3HB-*co*-3HV): mcl-PHA blend	10.4 ± 3.4	0	17

## Data Availability

The data presented in this study are available on request from the corresponding author.
